# Crystal Structure of *Kluyveromyces lactis* Glucokinase (*Kl*Glk1)

**DOI:** 10.3390/ijms20194821

**Published:** 2019-09-28

**Authors:** Krzysztof M. Zak, Magdalena Kalińska, Elżbieta Wątor, Katarzyna Kuśka, Rościsław Krutyhołowa, Grzegorz Dubin, Grzegorz M. Popowicz, Przemysław Grudnik

**Affiliations:** 1 Institute of Structural Biology, Helmholtz Zentrum München, Ingolstädter Landstrasse 1, 85764 Neuherberg, Germany; krzysztof.zak@helmholtz-muenchen.de (K.M.Z.); grzegorz.popowicz@helmholtz-muenchen.de (G.M.P.); 2 Malopolska Centre of Biotechnology, Jagiellonian University, ul. Gronostajowa 7a, 30-387 Krakow, Poland; guta@friend.pl (M.K.); elkon.wator@gmail.com (E.W.); kuska.kk@gmail.com (K.K.); rostyslav.krutyholova@gmail.com (R.K.); grzegorz.dubin@uj.edu.pl (G.D.); 3 Faculty of Biochemistry, Biophysics and Biotechnology, Jagiellonian University, ul. Gronostajowa 7, 30-387 Krakow, Poland; 4 Center for Integrated Protein Science Munich at Chair of Biomolecular NMR, Department Chemie, Technische Universität München, Lichtenbergstrasse 4, 85747 Garching, Germany

**Keywords:** *Kluyveromyces lactis*, glucokinase, sugar metabolism

## Abstract

Glucose phosphorylating enzymes are crucial in the regulation of basic cellular processes, including metabolism and gene expression. Glucokinases and hexokinases provide a pool of phosphorylated glucose in an adenosine diphosphate (ADP)- and ATP-dependent manner to shape the cell metabolism. The glucose processing enzymes from *Kluyveromyces lactis* are poorly characterized despite the emerging contribution of this yeast strain to industrial and laboratory scale biotechnology. The first reports on *K. lactis* glucokinase (*Kl*Glk1) positioned the enzyme as an essential component required for glucose signaling. Nevertheless, no biochemical and structural information was available until now. Here, we present the first crystal structure of *Kl*Glk1 together with biochemical characterization, including substrate specificity and enzyme kinetics. Additionally, comparative analysis of the presented structure and the prior structures of *lactis* hexokinase (*Kl*Hxk1) demonstrates the potential transitions between open and closed enzyme conformations upon ligand binding.

## 1. Introduction

Glucose is one of the main factors in metabolism and key regulators of gene expression in eukaryotic organisms, including yeast, protists, plants, and mammals. Glucose-dependent regulation requires glucose phosphorylation provided by glucose-phosphorylating enzymes, glucokinases, and hexokinases. These enzymes are responsible for intracellular trapping and metabolism initiation of monosaccharides and catalyze ATP-driven phosphorylation, yielding ADP and glucose-6-phosphate. Glucose phosphorylation is also considered as drug target against parasitic protists [1,2,3]. Additionally, some ADP-dependent sugar kinases were also characterized, but their role remains more elusive [4,5,6].

In yeast, plant and mammalian cells’ sugar kinases were shown to be responsible for glucose sensing and signaling [7,8,9,10], positioning those enzymes as providers of a carbon source and regulators of sugar-dependent cellular mechanisms. A number of hexokinases were demonstrated to translocate to the nucleus and intercede in the process of glucose repression of transcription in yeast [11] and plants [12]. In mammals, hexokinases have been shown to play a role in the mechanisms of sugar homeostasis [13]. Although the hexokinase interacting partners were shown to play a critical role in the activity of those enzymes [11,12,14,15], the limited structural information and lack of in vivo studies hinder the complete understanding of hexokinase role in nutrient signaling [16,17,18].

*Kluyveromyces lactis* is an emerging tool in biotechnology. Since the early 1990s, it has been employed as a host for recombinant protein expression for industrial use (summarized in [19]). One of the most important examples relates to its use in industrial production of lactase and bovine chymosin, enzymes which are important in food biotechnology [20]. Due to the growing importance of *K. lactis* in biotechnology, research on biology and metabolism of this microorganism gains value for both scientific and industrial reasons.

*Kl*Glk1 was reported for the first time by Kettner and colleagues [21]. The group identified the *KLLA0C01155g* gene in *K. lactis* genome on the basis of its high similarity to *Saccharomyces cerevisiae* glucokinase (*Sc*Glk1, nucleotide identity 62.4%) and glucokinase-like protein *Sc*EMI2 (nucleotide identity 63.3%) genes. Genomic analysis, followed by isolation and functional analysis of the protein, revealed its enzymatic activity and specificity. The *Kl*Glk1 mutants denoted the role of the enzyme as an accessory glucose phosphorylating protein (to *Kl*Hxk1) which might act as a glucose phosphorylation enzyme with currently unknown physiological function.

So far, *Kl*Hxk1 hexokinase is the only structurally characterized sugar metabolizing enzyme from *K. lactis* [22,23,24]. Here, we extend the structural understanding of *K. lactis* glucose phosphorylating enzymes. We have expressed and purified recombinant *Kl*Glk1 and solved its crystal structure at 2.6 Angstrom (Å) resolution. Comparative analysis of the structure of *Kl*Glk1 reported in this study and the prior structure of *Kl*Hxk1 demonstrates the transitions between open and closed enzyme conformations.

## 2. Results and Discussion

### 2.1. Biochemical Characterization

We have recombinantly expressed, purified, and crystalized *Kl*Glk1 glucokinase from *K. lactis* and characterized its enzymatic properties. First, we analyzed the protein’s thermal stability using both thermal shift assay (TSA) and Tycho analysis (Figure S1). Using these methods, the unfolding temperatures were determined at 41.9 ± 0.1 °C and 53.6 ± 0.05 °C for TSA and Tycho, respectively. Values obtained by TSA and Tycho cannot be directly compared due to different temperature gradients and readout methodology used by both techniques, although both document relatively low thermal stability of the protein.

**Figure 1:**
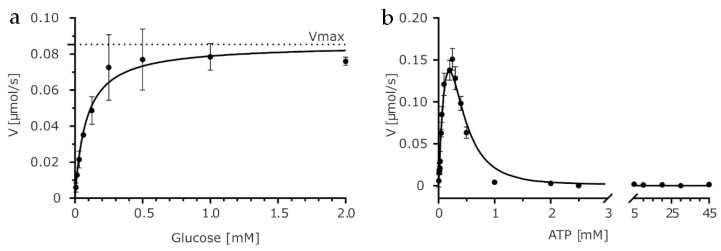
Kinetic analysis of *Kl*Glk1 activity. (**a**) Reaction velocity as a function of glucose concentration measured at 0.5 mM ATP. Solid line represents fit of standard Michaelis–Menten model, R^2^ = 0.98; V_max_ (0.085 ± 0.004 μM/s; dotted line), k_cat (app)_ (150 s^−1^) K_m_ (0.082 ± 0.013 mM). Error bars correspond to SD, N = 3. (**b**) Reaction velocity as a function of ATP concentration measured at 0.5 mM glucose. Substrate inhibition is clearly visible starting from 0.25 mM ATP. Solid line indicates a substrate inhibition model with a correction for dimerization, R^2^ = 0.97. Error bars correspond to SD, *N* = 3.

Next, we assayed the protein activity using constant ATP concentration equal to 0.5 mM and the standard Michaelis–Menten model to quantify the affinity (K_m_) for glucose (Figure 1A). Tested conditions yielded the apparent kinetic parameters k_cat (app)_ = 150 s^−1^ and K_m (app)_ = 0.08 mM.

In the previous work, Kuettner and colleagues observed that *K. lactis* JA6Δrag5 strain lacking *Kl*Hxk1 hexokinase revealed significant growth on glucose, but not on fructose. Thus, we tested *Kl*Glk1’s ability to catalyze phosphate transfer from ATP to fructose, and we observed no such activity at tested conditions.

Sugar kinases often exhibit inhibition by ATP. The phenomenon of substrate inhibition has been described for hexokinases which may be inhibited even by physiological concentrations of ATP [25]. Hence, we tested if *Kl*Glk1 is prone to substrate inhibition by ATP at a concentration above 0.25 mM (Figure 1B). We used fixed glucose concentration (0.5 mM) to determine the apparent kinetic parameters (k_cat (app)_ = 400 s^−1^, K_m (app)_ = 0.15 mM) of *Kl*Glk1 for ATP concentrations preceding substrate inhibition range. Comparative fitting of a standard Michaelis–Menten model with and without Hill coefficient (Figure S2A,B) revealed strong positive cooperativity at low ATP concentrations as indicated by the Hill coefficient of 1.97 determined from the V = Vmax*[ATP]^h^/(Km^h^ + [ATP]^h^) equation. Sigmoidal shape of the Hill plot (Figure S2C) suggests that *Kl*Glk1 exists in two states with different catalytic activity, with an apparent transition point at 45 μM ATP. At higher ATP concentrations, abnormally strong substrate inhibition takes place, which in pair with positive cooperativity observed in case of *Kl*Glk1 could be explained, among others, by protein dimerization. To fit the theoretical model to our data, we combined the classical Yoshino and Murakami substrate inhibition model of a complete type, which was previously validated on *Escherichia coli* phosphofructokinase II, with a kinetic model derived for dimerizing enzymes [26,27]. Interestingly, numerical values of k_cat_ and K_m_ calculated based on this model do not differ significantly from values estimated by classical Michaelis–Menten kinetics for a lower concentration range. The ratio of a substrate inhibition constant to a Michaelis–Menten constant (K_si_/K_m_) is close to 1.0, indicating a substrate inhibition of a complete type, which means that at high substrate concentrations, the [ES_1_S_2_]_2_ complex is formed, and it is unable to perform the reaction. As stated previously, strong substrate inhibition of *Kl*Glk1 may be explained, among others, by protein dimerization. Therefore, we investigated the possible dimerization using size-exclusion chromatography coupled to light scattering (right-angle light scattering/low-angle light scattering (RALS/LALS)). Analysis of RALS/LALS distribution demonstrated that *Kl*Glk1 elutes as a dimer of apparent molecular weight of 100 kDa (Figure S3). This observation goes in line with size-exclusion chromatography retention time calibrated with molecular weight standards.

### 2.2. Overall Structure

**Figure 2:**
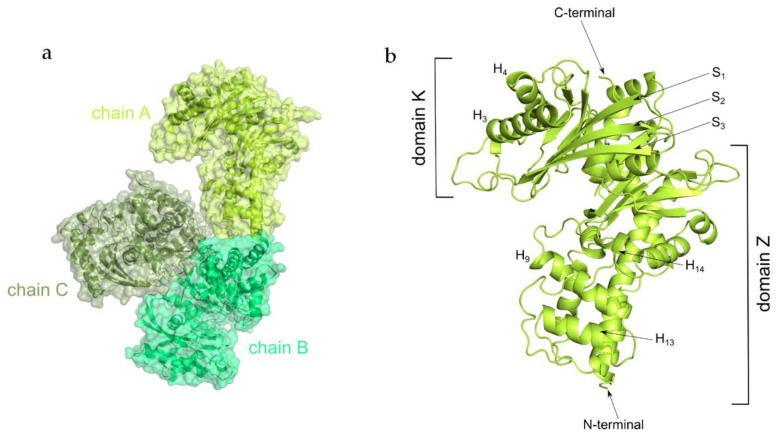
Crystal structure of *Kl*Glk1. (**a**) Arrangement of *Kl*Glk1 molecules in the asymmetric unit. (**b**) Architecture of *Kl*Glk1 monomer (chain A). H indicates helices, S indicates strands.

We crystallized *K*lGlk1 in an apo form. The crystal structure of *Kl*Glk1 was determined by molecular replacement pipeline MoRda using Protein Data Bank (PDB) entry 3O8M as a search model [28]. The structure was refined to R_work_/R_free_ values of 0.205/0.244 at 2.6 Å resolution (Table 1). The asymmetric unit contains three KlGlk1 molecules and the unit cell belongs to C 2 2 2_1_ space group (Figure 2a).

*Kl*Glk1 consists of two ribonuclease H-like type domains (Figure 2b, Figures S4 and S5). The N-terminal, smaller domain K represents compact fold with beta strands in the domain’s core and short alpha helices located between strands 1–3 and 10–13 as well as at the top of domain K (helices 3 and 4). Larger domain Z consists of central mixed β-sheet (strands 10–13) flanked by alpha helices connected by the extended loops resulting in the elongated shape of the domain. Inspection of the overlap among the molecules contained in the asymmetric unit (ASU) does not indicate any significant structural differences as confirmed by low root mean square deviation (RMSD) values calculated by TM-align (RMSD calculated based on backbone C alpha atoms; RMSD values of backbone atoms of chains A/B, B/C and A/C are 0.53 for 469 residues, 0.62 for 469 residues, 0.54 for 470 residues, respectively) [29]. Two out of three *Kl*Glk1 monomers (chains B and C) create relatively large contacts in the crystal, resulting in a total buried area of 372.6 Å^2^ (interaction surfaces were calculated using the PISA server) [30]. The third molecule (chain A) creates less significant contacts only with monomer B, resulting in a total buried area of 87.1 Å^2^. Nonetheless, this analysis of the arrangement of molecules in the crystal does not indicate stable dimerization—the buried surface areas are too small to ensure stable dimerization in solution. This is confirmed by automated analysis criteria implemented in the PISA server, suggesting no formation of stable quaternary structures, thereby indicating protein’s monomeric state in the presented crystal structure [30].

### 2.3. Structural Comparison between KlGlk1 and KlHxk1 Glucokinases

*Kl*Hxk1, the closest homolog of *Kl*Glk1, was crystallized in several different crystal forms showing the monomeric and dimeric states of the enzyme [23,24]. The structures of dimeric *Kl*Hxk1 show that protein forms a symmetrical, ring-shaped homodimer with a head-to-tail arrangement of the protein molecules. The structures of apo and substrate (glucose) bound forms provided molecular insight into the mechanism of substrate binding and related conformational changes.

Comparative analysis of the *Kl*Glk1 structure and available *Kl*Hxk1 Protein Data Bank (PDB) entries presenting the later protein in open state shows significant similarities in the overall structure despite low amino acid sequence identity (Figure S6). Superposition of *Kl*Glk1 and *Kl*Hxk1 in open state (PDB 3O08) demonstrate almost identical fold and position of both domains. Only small differences are noticeable in the loop regions between residues 135–145, 330–347, and 412–422, most probably due to the flexible nature of these parts characterized by high B factors (Figure S7). More pronounced differences are visible in the 246–266 region, where *Kl*Hxk1 folds into a helix flanked by unstructured regions compared to the long, unstructured loop in the *Kl*Hxk1, as well as between 450–462 residues of *Kl*Glk1 where the loop region with an inserted short helix is significantly shorter in *Kl*Glk1 than in *Kl*Hxk1 (*Kl*Glk1 residues 450–462 corresponds to residues 437–453 of *Kl*Hxk1).

**Figure 3:**
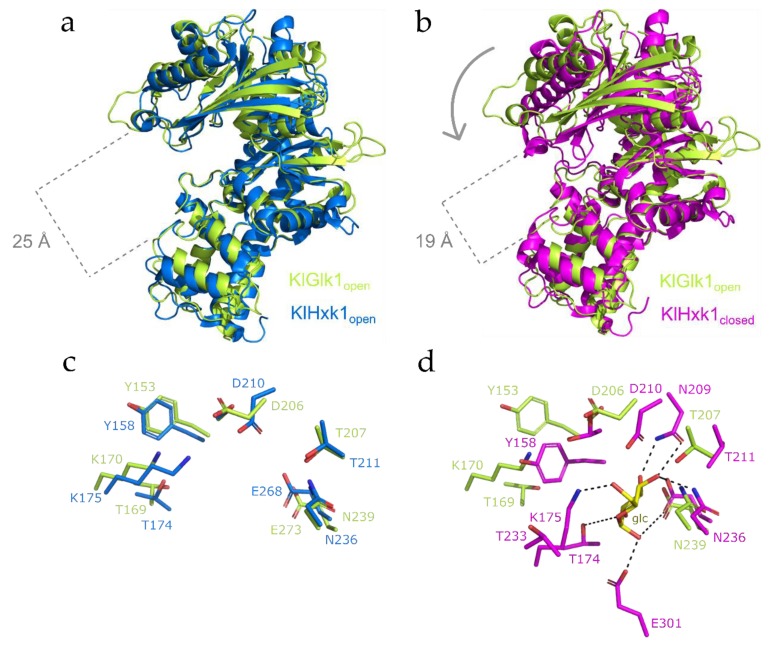
Comparison of crystal structures of *Kl*Glk1 and *Kl*Hxk1 kinases. (**a**) Superposition of *Kl*Glk1 and KlHxk1 (PDB:3o08) in open states. (**b**) Superposition of *Kl*Glk1 in the open state and *Kl*Hxk1 in the closed state (PDB:3o8m). (**c**) Superposition of active site residues of *Kl*Glk1 and *Kl*Hxk1 in open state (PDB:3o08). (**d**) Superposition of active site residues of *Kl*Glk1 and *Kl*Hxk1 in closed state (PDB:3o8m). Superpositions were made over large domains of *Kl*Glk1 and *Kl*Hxk1 (TM-align module in PyMol software, residues 2–60 and 220–481 for *Kl*Glk1, residues 15–60 and 220–484 for *Kl*Hxk1).

The crystal structures of *Kl*Hxk1 reported previously indicate that hexokinase undergoes profound conformational changes upon substrate binding [23]. Superposition of the structure of *Kl*Glk1 reported in this study and the structure of *Kl*Hxk1 in a closed, substrate bound conformation (PDB 3O8M; Figure 3) suggests that *Kl*Glk1 structure has been determined in its open state (accordingly, no substrate is present at the active site). Superposition and detailed comparison shows that the relative arrangement of small and large domains in *Kl*Glk1 and substrate bound *Kl*Hxk1 is different. In *Kl*Hxk1, the substrate binding promotes the closed conformation of protein, whereas apo *Kl*Glk1 remains in open state. This phenomenon is typical for sugar phosphorylating proteins and has been described before [5,31]. The difference in protein conformation and domain location supports our suggestion concerning *Kl*Glk1 open state. Moreover, the protein part undergoing conformational change encompasses the region of 115–195 residues that folds into helices H_3_ and H_4_ and stands S_4_–S_7_ with a 165–174 loop that might be responsible for stabilizing the substrate at the active site of the enzyme.

We report here a *Kl*Glk1 crystal structure in an open conformation. The kinetic assays performed on recombinant protein confirmed protein activity towards glucose and no activity towards fructose. We also observed a relatively strong substrate inhibition by ATP in the concentration range exceeding 0.25 mM. This indicates a tight regulation of *Kl*Glk1 by ATP. The protein remains inactive when the ATP level in the cell is high, which suggests that *Kl*Glk1 serves as a safety mechanism preventing too fast energy production. Both kinetic analysis and light scattering studies show that *Kl*Glk1 forms a dimer in solution, which is likely related to activity regulation by ATP.

## 3. Materials and Methods

### 3.1. Cloning, Expression and Purification

The *Kluyveromyces lactis* KLLA0C01155g gene was codon optimized for improved expression in *Escherichia coli.* The gene was synthesized (GenScript, Leiden, Netherlands) and subcloned to a pET24d expression vector. N-terminal His-tagged *Kl*Glk1 was expressed in *E. coli* BL21 (DE3) using terrific broth medium supplemented with kanamycin. Briefly, cells were cultivated until OD_600_ value of 1 at 37 °C, the expression was induced with 0.5 mM isopropyl-β-d-thiogalactoside (IPTG), and cultures were incubated for 16 h at 16 °C. After expression, cells were collected and resuspended in lysis buffer containing 150 mM NaCl, 20 mM imidazole, 20 mM (4-(2-hydroxyethyl)-1-piperazineethanesulfonic acid) (HEPES) pH 8.0 and lysed by sonication. Soluble fraction was loaded onto Ni-Sepharose (GE Healthcare, Uppsala, Sweden) column equilibrated with lysis buffer. Protein was eluted with lysis buffer containing 500 mM imidazole and mixed with Tobacco etch virus (TEV) protease. After incubation, solution was loaded onto Ni-Sepharose resin to remove His-tag and TEV protease. Finally, *Kl*Glk1 was concentrated and purified to homogeneity by gel filtration using Superdex 75 (GE Healthcare, Uppsala, Sweden) in 150 mM NaCl, 20 mM HEPES, pH 8.0 (Figure S8). Fractions containing protein of interest were pooled and concentrated to 10 mg/mL.

### 3.2. Crystallization and Structure Determination

Crystallization screening was carried out using commercially available buffer sets (Molecular Dimensions) in a sitting-drop vapor diffusion setup by mixing 1 μL of protein solution and 1 μL of buffer solution at room temperature. Diffraction quality crystals were obtained from condition containing 0.15 M potassium bromide and 30% (*w/v*) poly(ethylene glycol) (PEG) monomethyl ether (MME) 2000. Crystals were cryoprotected with 30% ethylene glycol in mother liquor and flash frozen in liquid nitrogen.

Diffraction data were collected at 14.1 beamline at BESSY II, Berlin, Germany [32] using Pilatus 6M detector to 2.6 Å resolution (Table 1). The data were indexed and integrated using XDS [33] then scaled and merged using Scala [34]. Structure was solved using MoRda server [28] and Phaser [35] using PDB 3O8M as a search model. As the search model shared only 38% of its amino acid sequence identity with *Kl*Glk1, several parts of the model were manually rebuilt using *Coot* [36]. Restrained refinement was performed using Phenix [37]. Five percent of the reflections were used for cross-validation analysis, and the behavior of the R_free_ was employed to monitor the refinement strategy. Water molecules were added using Coot and subsequently manually inspected. The loop containing residues 43–48 (chain A; 43–49 for chain B) was not fully resolved in the electron density map and therefore was not modeled. The final model comprises residues 2–481 from each monomer and 196 water molecules.

### 3.3. Activity Assay

*Kl*Glk1 activity was assessed by monitoring the level of one of the reaction products (glucose-6-phosphate) in a coupled glucose-6-phosphate dehydrogenase (G6PD) reaction essentially as described previously [5,6]. Unless stated otherwise, the assays were performed at 37 °C in reaction buffer (20 mM Tris-HCl pH 7.4, 250 mM sucrose, 50 mM KCl, 5 mM MgCl_2_) supplemented with glucose-6-phosphate dehydrogenase (1 unit) and nicotinamide adenine dinucleotide phosphate (NADP) (0.5 mM). The *Kl*Glk1 protein concentration was adjusted to 0.16 mg/mL (3 µM).

### 3.4. RALS/LALS Analysis

The oligomeric state of *Kl*Glk1 was investigated by size-exclusion chromatography (Superdex 200 Increase 10/300 GL column) coupled to right-angle light scattering (RALS) followed by measurement of refractive index (RI) using OMNISEC REVEAL. Measurements were performed in 50 mM Tris pH 7.8, 200 mM NaCl, 5 mM β-ME buffer, and results were analyzed using OMNISEC software.

### 3.5. Protein Stability Analysis

Melting points (Tm-values) of *Kl*Glk1 glucokinase were obtained using thermal shift assay (TSA) and real time monitoring of changes in the intrinsic protein fluorescence by Tycho NT.6 [38]. For TSA analysis, *Kl*Glk1 (1 mg/mL) was incubated with Sypro Orange dye in 150 mM NaCl, 20 mM HEPES, pH 8.0. Fluorescence signal of Sypro Orange was determined as a function of temperature between 5 and 95 °C in increments of 1.2 °C/min. The melting temperature was calculated as the inflexion point of the fluorescence vs. temperature function. Each experiment was carried out in triplicate. For Tycho NT.6 analysis, capillary was loaded with 10 µL of *Kl*Glk1 (1 mg/mL) in 150 mM NaCl, 20 mM HEPES, pH 8.0 and heated from 35 to 95 °C in 3 min overall time. The melting temperature was calculated as the inflexion point of the 350 nm/330 nm intrinsic fluorescence vs. temperature function.

### 3.6. Protein Data Bank Accession Code

Coordinates and structure factors for the *Kl*Glk1 have been deposited in Protein Data Bank (PDB) with code 6R2N.

## 4. Conclusions

Our study expands the understanding of glucose metabolizing enzymes in *K. lactis* by presenting the first crystal structure of its unique glucokinase. The *Kl*Glk1 structure along with the biochemical data help to understand the mechanisms responsible for glucose sensing and signaling, therefore allowing for more intensive use of *K. lactis* for both scientific and industrial applications.

## Figures and Tables

**Table 1:** Data collection and refinement statistics.

	***Kl*Glk1 (PDB 6R2N)**
**Data Collection**	
Space group	C 2 2 2_1_
Total reflections/Unique reflections	139134 (13371)
Cell dimensions	102.0 Å, 122.7 Å, 360.3 Å 90^0^, 90^0^, 90^0^
Wavelength (Å)	0.9184
Resolution (Å)	49.08-2.596 (2.689-2.596)
Mean I/sigma (I)	13.6 (1.7)
Completeness (%)	99.58 (97.10)
Multiplicity	2.0 (2.0)
CC_1/2_	98.6 (52.8)
**Refinement**	
Reflections used in refinement	69952 (6762)
Reflections used for R-free	1099 (107)
R-work	0.2045 (0.3300)
R-free	0.2396 (0.3345)
**Number of Atoms/Molecules**	
non-hydrogen atoms	11273
macromolecules	11032
ligands^♦^	45
solvent	196
**B-factors**	
average B-factor (Å)	76.69
macromolecules (Å)	77.06
ligands (Å)	86.48
solvent (Å)	53.66
wilson B-factor (Å)	53.71
**Ramachandran plot**	
Ramachandran favored (%)	96.88
Ramachandran allowed (%)	3.05
Ramachandran outliers (%)	0.07
**R. m. s. deviations**	
Bonds (Å)	0.004
Angles (°)	0.96
Rotamer outliers (%)	0.92
Clashscore	3.08

Values in parentheses are for high resolution shell. ♦ Ligands in the presented structure include chemicals from crystallization condition and cryoprotectant solution and are not biologically relevant in the presented context.

## Supplementary Materials

Supplementary materials can be found at https://www.mdpi.com/1422-0067/20/19/4821/s1.

## Abbreviations

ATP	Adenosine triphosphate
ADP	Adenosine diphosphate
Å	Angstrom
TSA	Thermal Shift Assay
RMSD	Root Mean Square Deviation
IPTG	Isopropyl-β-D-thiogalactoside
HEPES	(4-(2-hydroxyethyl)-1-piperazineethanesulfonic acid)
PEG	Poly(ethylene glycol)
RALS/LALS	Right-angle light scattering/low-angle light scattering
TRIS	Trisaminomethane
NADP	Nicotinamide adenine dinucleotide phosphate
