# Supplementary material for: Crystal Structure of Kluyveromyces lactis Glucokinase (KlGlk1)
Source: Int J Mol Sci. 2019 Sep 28;20(19):4821. doi: 10.3390/ijms20194821 (PMC6801647; doi:10.3390/ijms20194821)
Supplement: Supplementary file 1 [file ijms-20-04821-s001.pdf]

International Journal of
Molecular Sciences

MDPI

Supplemental Information

Supplemental Figures

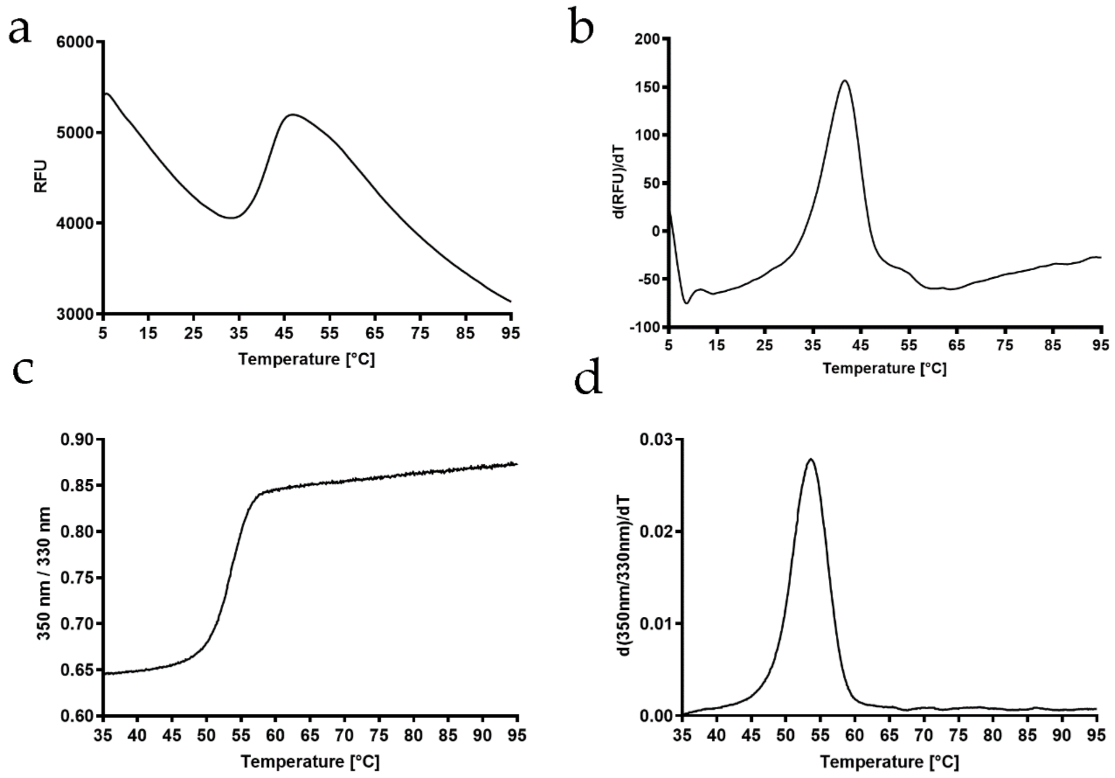

Figure S1. Stability studies of *Kl*Glk1 protein using Thermal Shift Assay (panels **a** and **b**) and Tycho (panels **c** and **d**). Panels (**a)** and (**c)** represent the raw fluorescence data, panels (**b)** and (**d)** show the corresponding first derivative.

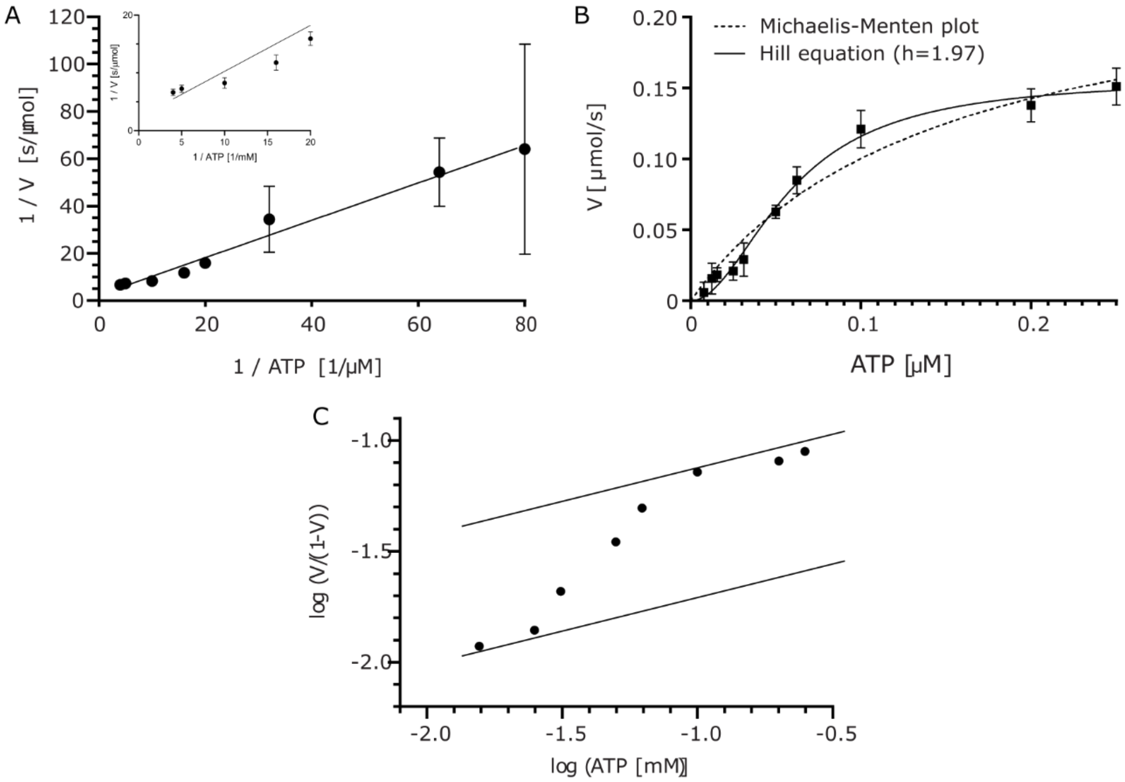

Figure S2. Kinetic analysis of *Kl*Glk1 activity. (**a**) Lineweaver-Burk linearization (reciprocal plot). Datapoints for low ATP concentrations obey linear trend and were considered for fitting of a linear model (solid line, R^2=0.98). V_{max} was determined from the Y-intercept, X-intercept is equal to -1/Km. Error bars correspond to SD, N=3. (**b**) Comparative fit of classical Michaelis-Menten plot (R^2=0.96) and a Hill plot (R^2=0.99, h=1.97). (**c**) Hill Plot. Error bars correspond to SD, N=3.

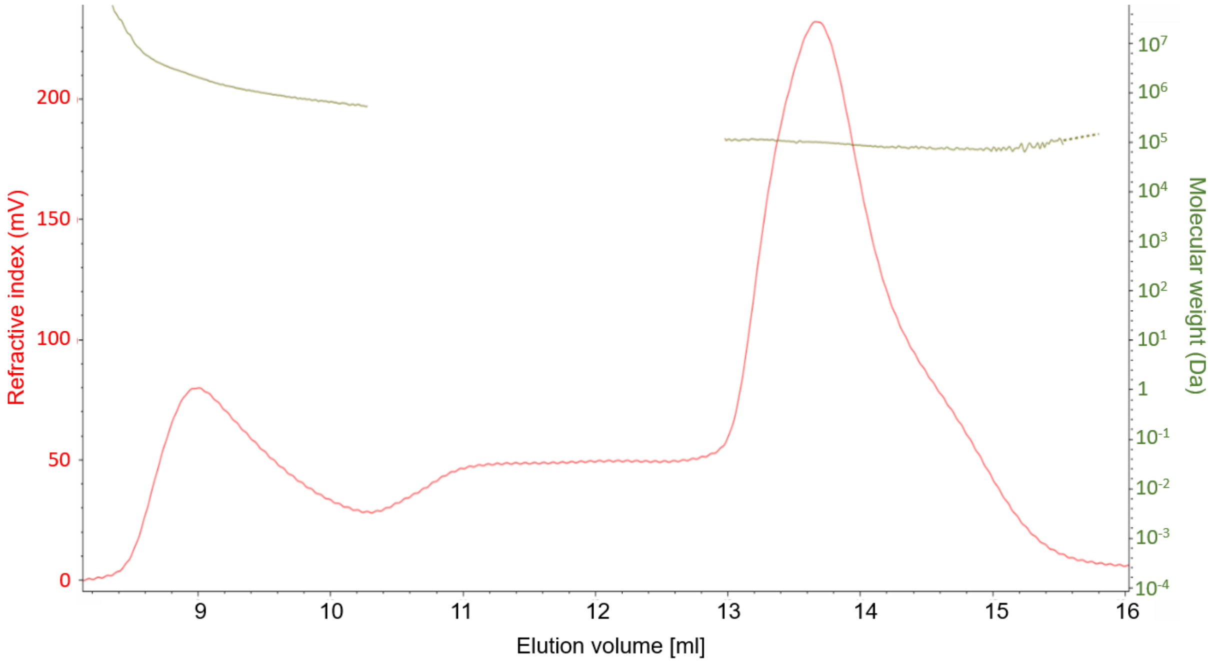

Figure S3. Analysis of RALS/LALS distribution for *Kl*Glk1. Elution profile indicates formation of a *Kl*Glk1 dimer in the solution with calculated molecular weight of 100 kDa (second peak). Refractive index (mV) and corresponding calculated molecular weight (Da) are represented as a red and green line, respectively.

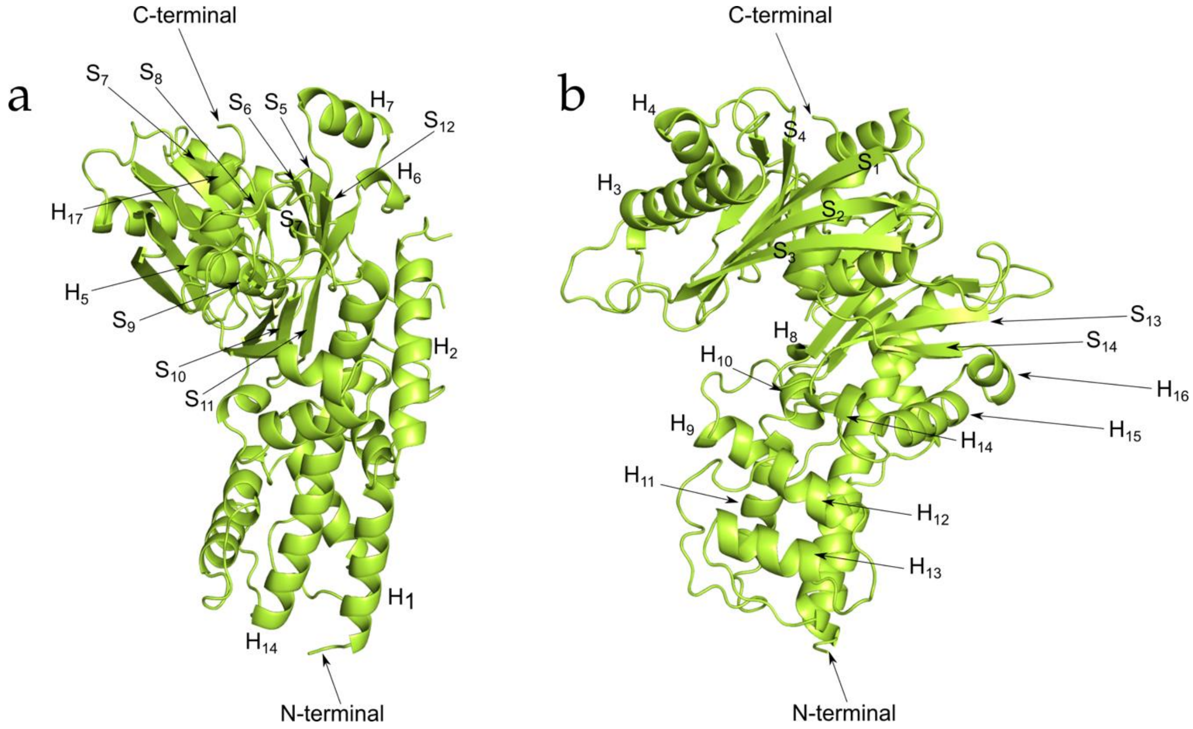

Figure S4. Complete topology of *Kl*Glk1 protein. Side view (**a**) and front view (**b**) of *Kl*Glk1. H indicates helices, S indicates strands.

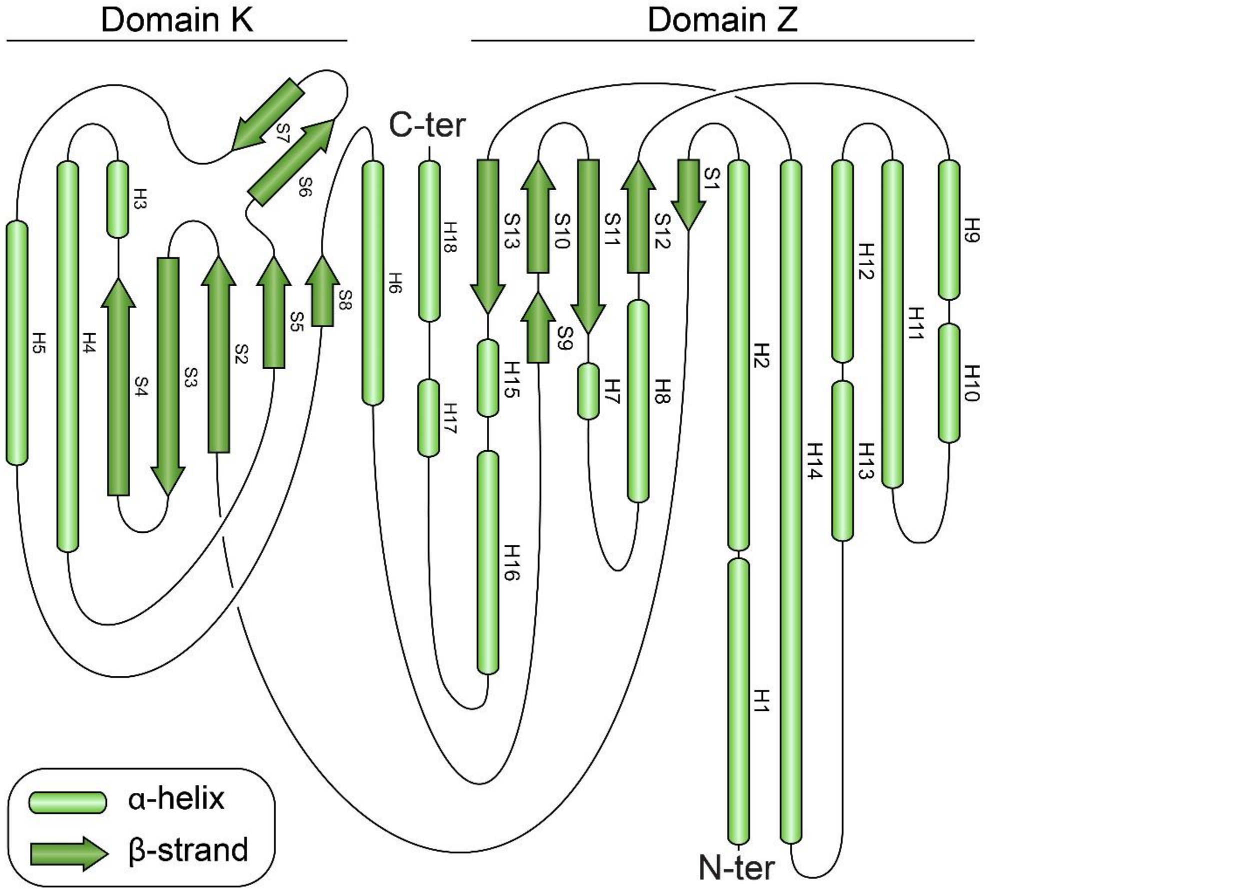

Figure S5. 2D topology map of *Kl*Glk1 monomer.

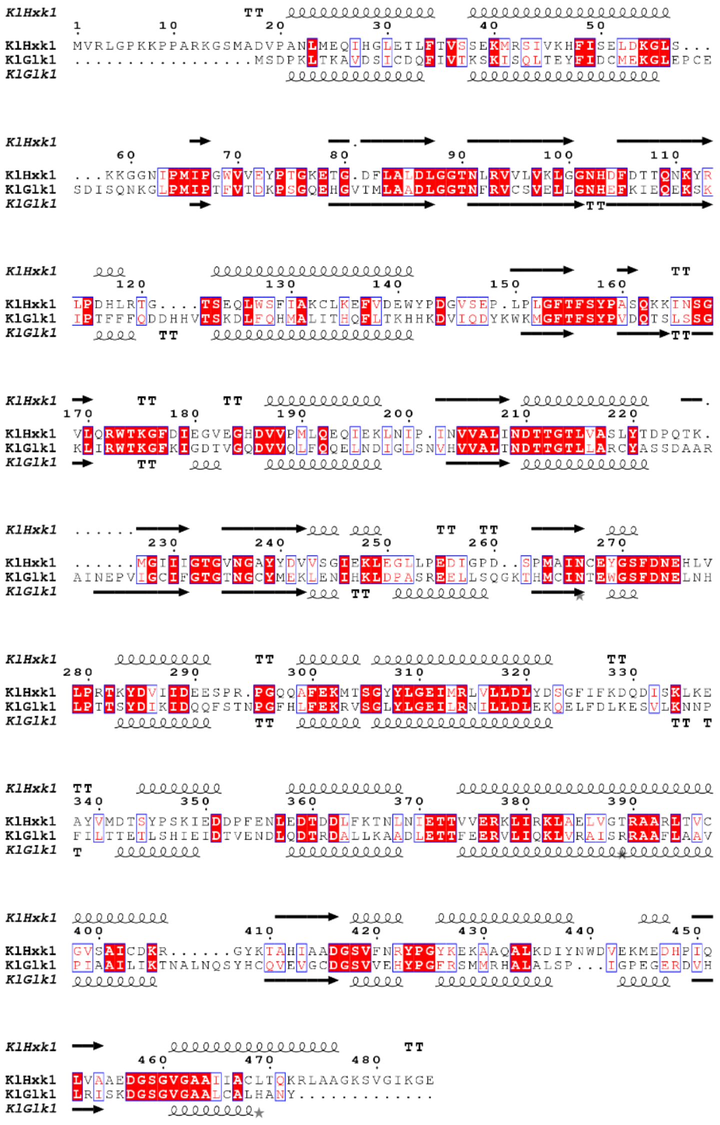

Figure S6. Amino acid sequence alignment of *Kl*Glk1 and KlHxk1 glucose kinases from *Kluyveromyces lactis*. Amino acid sequence identity between *Kl*Glk1 and KlHxk1 is 37%. Sequence Secondary structures for both proteins are indicated.

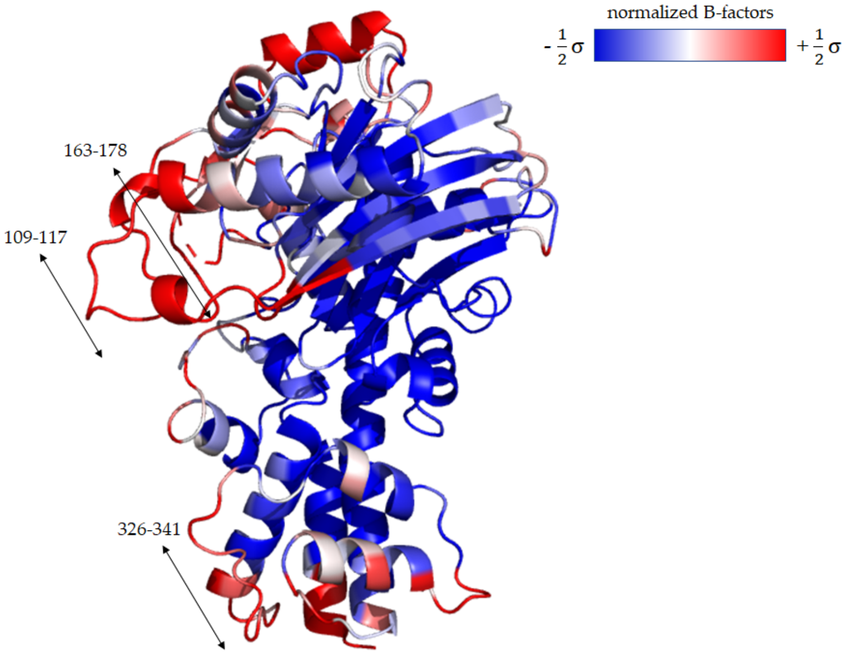

Figure S7. Coloured representation of normalized B-factors in *Kl*Glk1 protein structure. Red parts represent high, white middle, blue low B factors.

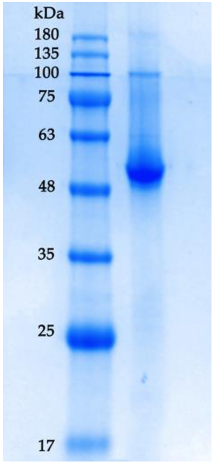

Figure S8. SDS-PAGE gel showing purity of the *Kl*Glk1 after final step of purification.
